# What Are the Driving Forces of Urban CO_2_ Emissions in China? A Refined Scale Analysis between National and Urban Agglomeration Levels

**DOI:** 10.3390/ijerph16193692

**Published:** 2019-09-30

**Authors:** Hui Wang, Guifen Liu, Kaifang Shi

**Affiliations:** 1 School of Geographical Sciences, State Cultivation Base of Eco-agriculture for Southwest Mountainous Land, Southwest University, Chongqing 400715, China; whh123456@email.swu.edu.cn; 2 Chongqing Jinfo Mountain Field Scientific Observation and Research Station for Kaster Ecosystem, School of Geographical Sciences, Southwest University, Chongqing 400715, China; 3 Chongqing Engineering Research Centre for Remote Sensing Big Data Application, School of Geographical Sciences, Southwest University, Chongqing 400715, China; 4 Shandong Provincial Eco-environment Monitoring Center, Jinan 250000, China; jnhbjliuguifen@jn.shandong.cn

**Keywords:** urban CO_2_ emissions, driving forces, nighttime light data, multiscale analysis, China

## Abstract

With the advancement of society and the economy, environmental problems have increasingly emerged, in particular, problems with urban CO_2_ emissions. Exploring the driving forces of urban CO_2_ emissions is necessary to gain a better understanding of the spatial patterns, processes, and mechanisms of environmental problems. Thus, the purpose of this study was to quantify the driving forces of urban CO_2_ emissions from 2000 to 2015 in China, including explicit consideration of a comparative analysis between national and urban agglomeration levels. Urban CO_2_ emissions with a 1-km spatial resolution were extracted for built-up areas based on the anthropogenic carbon dioxide (ODIAC) fossil fuel emission dataset. Six factors, namely precipitation, slope, temperature, population density, normalized difference vegetation index (NDVI), and gross domestic product (GDP), were selected to investigate the driving forces of urban CO_2_ emissions in China. Then, a probit model was applied to examine the effects of potential factors on urban CO_2_ emissions. The results revealed that the population, GDP, and NDVI were all positive driving forces, but that temperature and precipitation had negative effects on urban CO_2_ emissions at the national level. In the middle and south Liaoning urban agglomeration (MSL), the slope, population density, NDVI, and GDP were significant influencing factors. In the Pearl River Delta urban agglomeration (PRD), six factors had significant impacts on urban CO_2_ emissions, all of which were positive except for slope, which was a negative factor. Due to China’s hierarchical administrative levels, the model results suggest that regardless of which level is adopted, the impacts of the driving factors on urban CO_2_ emissions are quite different at the national compared to the urban agglomeration level. The degrees of influence of most factors at the national level were lower than those of factors at the urban agglomeration level. Based on an analysis of the forces driving urban CO_2_ emissions, we propose that it is necessary that the environment play a guiding role while regions formulate policies which are suitable for emission reductions according to their distinct characteristics.

## 1. Introduction

With the advance of global socioeconomics, greenhouse gas emissions are continuously increasing [1,2]. It is generally known that greenhouse gas emissions have significant negative impacts on the sustainable development of humans worldwide [3,4]. Among the many greenhouse gases, carbon dioxide (CO_2_) is particularly highlighted. CO_2_ emissions from energy consumption related to human activities (CO_2_ emissions for short) could promote global gross CO_2_ emissions, thus exacerbating the carbon imbalance, which could pose an enormous threat to global sustainable development by causing catastrophic and irreversible damage [3,5,6], such as the melting of glaciers, droughts, flooding, and mudslides [7,8]. Many scholars have gradually recognized that as the centers of human activities, urban areas could play an important role in CO_2_ emissions [9,10,11]. The International Energy Agency has reported that urban areas consume 67% of all the energy produced globally, and account for 71% of global CO_2_ emissions [12,13]. Therefore, it is necessary and important to study urban CO_2_ emissions, especially in rapidly-developing countries.

The economy of China, which is the largest developing country, has boomed since initiating its reforms and opening up. In conjunction with this boom, all aspects of CO_2_ emissions have increased dramatically, including human electrical CO_2_ emissions [14,15], residential CO_2_ emissions [16], and other CO_2_ emissions [17]. According to international statistics, China has been the largest CO_2_ emitter since 2006 [18]. In addition, due to the demand of societal and economic development, the amount of land used for urban construction has rapidly increased in recent years. Urban areas in China exhibited an increasing trend in the period of 1992–2015, from 4.88 × 10^3^ km^2^ to 31.32 × 10^3^ km^2^ [19,20]. The urbanization level in China has exceeded the world average since 2013, reaching 58.52% in 2017 [21], and thereby accelerating the development of CO_2_ emissions [22,23]. Because rapid urbanization has promoted high CO_2_ emissions, maintaining urban development while effectively reducing urban CO_2_ emissions poses a great challenge for China [24,25].

In view of the importance and urgency of CO_2_ emission reduction in China, and the lack of relevant policies and measures, policymakers and academia are concerned about how to implement reasonable low-carbon planning and design policies for urban CO_2_ emissions [17,26,27]. To better solve these problems and offer more comprehensive solutions, first and foremost, we should determine the driving forces of urban CO_2_ emissions, such as the forces that will drive a reduction in urban CO_2_ emissions and those that will increase urban CO_2_ emissions. Understanding the driving forces could help us to determine the factor sources of CO_2_ emissions [28,29] which will help to reduce urban CO_2_ emissions [30]. Many studies have attempted to explore the driving forces of urban CO_2_ emissions, aiming to finally achieve the goal of energy conservation and CO_2_ emission reductions. For example, Shi et al. [17] found that urbanization rate, population, and gross domestic product (GDP) played important roles in urban CO_2_ emissions in China at the city level. Shi et al. [10] also indicated that urban CO_2_ emissions had significant positive correlations with population and GDP in China at the national, regional, and urban agglomeration scales. Bai et al. [19] analyzed the impact of changes in consumption patterns on household CO_2_ emission increments, electricity, and transportation factors. The extended log-mean Divisia index method based on the Kaya identity was adopted by Wang et al. [31] to examine the main driving forces of CO_2_ emissions in both the industrial and residential sectors. In addition, Wang et al. [32] calculated CO_2_ emissions from both urban and rural residential consumption in the Beijing-Tianjin-Hebei region by applying an input–output model. Wen et al. [33] analyzed the driving forces in China’s Yangtze River Economic Zone using pertinent data, including per capita GDP, energy efficiency, urban level, and industry and energy structures. Wang et al. [34] examined the effects of socioeconomic factors, urban form, and transportation factors, and applied an econometric model and a comprehensive panel dataset for four Chinese megacities—Beijing, Tianjin, Shanghai, and Guangzhou. To examine the influences of population scale, income level, population density, and price of house-based residential energy consumption and CO_2_ emissions, Miao et al. [35] applied an extended stochastic impacts by regression on population, affluence, and technology (STIRPAT) model based on city-level data. Although there have been many studies on the driving forces of urban CO_2_ emissions from different perspectives, most studies have focused on the potential factors at the scale of the administrative region, which assigns a value of urban CO_2_ emissions to the entire administrative region and ignores the differences in that region’s internal structures [10,36]. Within many administrative regions, areas exist that are not sources of CO_2_ emissions, for example, bodies of water and large national scenic areas [37,38]. Thus, it is necessary to explore the driving forces of urban CO_2_ emissions on a refined scale.

Due to differences in the environmental and socioeconomic conditions across China, different regions have formed with great disparities in urban development and CO_2_ emissions [39]. Some empirical studies have quantified the driving forces of urban CO_2_ emissions in different geographical areas in China. For example, Wang et al. [40] evaluated the CO_2_ emission efficiency in the Pearl River Delta and indicated that compact urban development could help to improve CO_2_ emission efficiency. Li et al. [41] explored the effects of urban forms on CO_2_ emissions in 288 cities in China. Zhao et al. [42] applied nighttime light datasets and spatial econometric models to examine the socioeconomic and climatic factors associated with spatiotemporal CO_2_ emissions by dividing China into four parts. Feng et al. [43] applied a system dynamics model to model the energy consumption and CO_2_ emission trends for the city of Beijing. However, evaluation of the driving forces of urban CO_2_ emissions has mostly been conducted in specific regions, and regional or scale differences in urban CO_2_ emissions have rarely been discussed. Generally, it is not appropriate to transfer findings from one spatial scale to another, because socioeconomic development, e.g., urban CO_2_ emissions, is sensitive to scale changes [17,44]. Scale and hierarchy evaluation are very significant for a better understanding of the complexity of China’s regional differences in urban CO_2_ emissions [45]. Although many studies have examined the driving forces of CO_2_ emissions in a number of cities, regions, or counties, an evaluation of the driving forces of urban CO_2_ emissions based on a sampling approach on multiple scales, which is necessary for government policymakers, is still lacking.

This study aims to explore the driving forces of urban CO_2_ emissions in China. The contributions of this study are summarized as follows:

(1) quantifying the driving forces of urban CO_2_ emissions at a finer spatial resolution (e.g., 1-km spatial resolution) using multiple-source data from 2000 to 2015;
(2) evaluating the driving forces of urban CO_2_ emissions from multiple spatial levels;
(3) comparing regional differences in the driving forces of urban CO_2_ emissions.

To address the above questions, we conducted experiments at two administrative levels (i.e., the national and urban agglomeration levels) to test our evaluation in this study. First, urban CO_2_ emissions and potential driving force data were extracted from multiple-source data from 2000 to 2015. Second, the probit model was employed to evaluate the driving forces of urban CO_2_ emissions on the national and urban agglomeration levels. Third, the differences between the national and urban agglomeration levels and between the different urban agglomerations were analyzed and compared, according to the factor relations of the urban agglomerations.

The remainder of the study was organized as follows. The second section introduces the data and methods. The third section presents the results. A discussion is presented in the fourth section, and the last section describes the conclusions and policy implications.

## 2. Data and Methods

### 2.1. Study Areas

**Figure 1:**
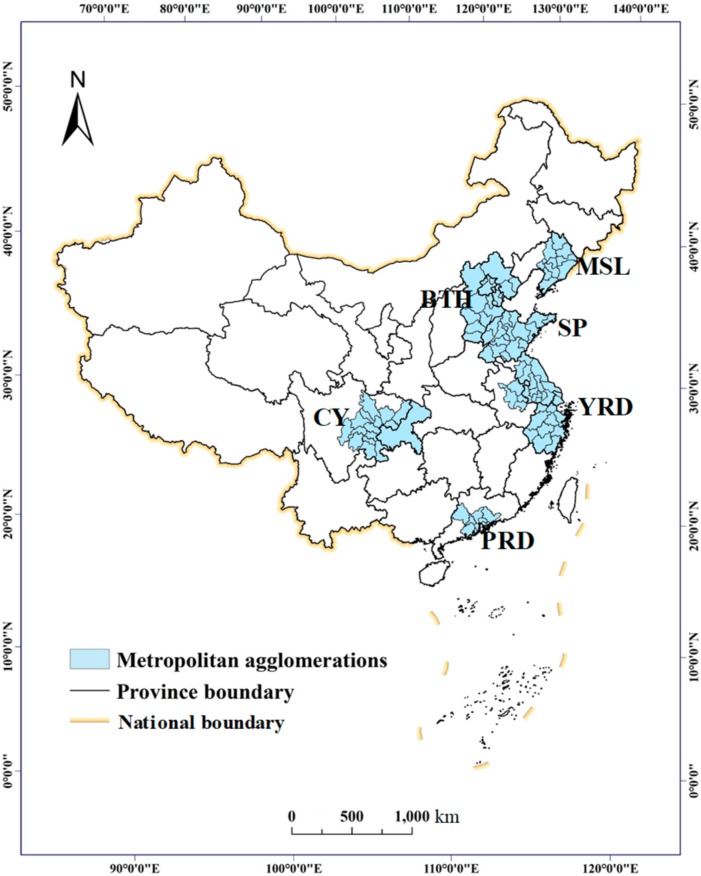
Spatial distribution of the study areas. Note: BTH represents the Beijing-Tianjin-Hebei urban agglomeration; MSL represents the middle and south Liaoning urban agglomeration; SP represents the Shandong Peninsula urban agglomeration; CY represents the Chengdu-Chongqing urban agglomeration; YRD represents the Yangtze River Delta urban agglomeration; and PRD represents the Pearl River Delta urban agglomeration.

To effectively quantify and compare the driving forces of urban CO_2_ emissions in China, study areas were selected from national and urban agglomeration levels for a multiscale analysis (Figure 1). The main justification for selecting these is that most previous, related, urban CO_2_ emissions studies have been analyzed on these levels [46,47,48]. Specifically, at the urban agglomeration level, six urban agglomerations were selected as study areas, namely, the Beijing-Tianjin-Hebei urban agglomeration (BTH), the middle and south Liaoning urban agglomeration (MSL), the Shandong Peninsula urban agglomeration (SP), the Chengdu-Chongqing urban agglomeration (CY), the Yangtze River Delta urban agglomeration (YRD), and the Pearl River Delta urban agglomeration (PRD). BTH is characterized by an industrial population agglomeration area, especially in Beijing, and is a megacity with extremely high population density, so urban CO_2_ emissions may be very high. MSL is an old heavy-industry area, and SP has many state-owned enterprises. CY is a representative western urban agglomeration. YRD, as a traditional economic development zone, has developed automobile, chemical, and other industries for decades which have caused high urban CO_2_ emissions. PRD is characterized by light industry and foreign trade. These six urban agglomerations are the most developed, densely populated, and economically-active areas in China and contain almost all the characteristics of China’s urban agglomerations (Figure 1). Specifically, these urban agglomerations in total have a population of 499,830,000, which is approximately 36% of the whole population according to the China Statistical Yearbook of 2018. These urban agglomerations have areas of 1,003,418 km^2^, representing approximately 10% of China’s territory [24]. Although the economic value created by these areas is considerable, they also suffer from tremendous problems, such as greenhouse gas effects, water pollution, and air pollution. CO_2_ emissions are rising annually and are more than 8×10^8^ t. Therefore, it is necessary to study the CO_2_ emission levels and the factors affecting them in these regions.

### 2.2. Urban CO_2_ Emissions

Accurately extracting urban CO_2_ emissions is a prerequisite for evaluating the driving forces of urban CO_2_ emissions in China. In this study, the extraction of urban CO_2_ emissions is divided into three steps. First, urban areas were extracted; then, data on China’s CO_2_ emissions were obtained. Finally, CO_2_ emissions for each urban area were estimated.

**Figure 2:**
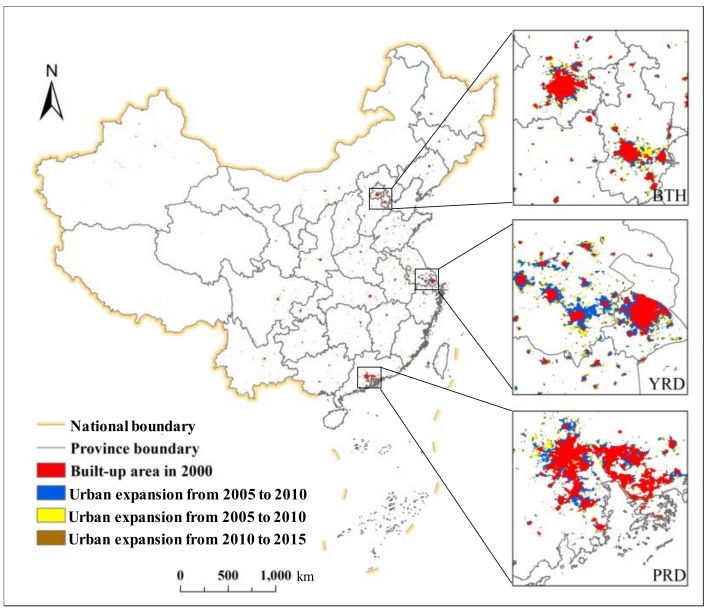
Urban expansion in China from 2000 to 2015. Note: The red represents urban areas in 2000; the blue represents urban areas in 2005; the yellow represents urban areas in 2010; and the brown represents urban areas in 2015. The three magnified urban agglomeration areas are BTH, the YRD, and the PRD.

At present, there are many methods for extracting urban areas. Nighttime light data have been shown to provide an effective way to extract urban areas on a large scale [49,50]. Most previous studies used two types of raw remote sensing data to extract urban areas, namely, the US Air Force Defense Meteorological Satellite Program’s Operational Linescan System (DMSP-OLS) data, and the National Polar-orbiting Partnership (NPP)-Visible Infrared Imaging Radiometer Suite (VIIRS) data. However, the main problem is determining the threshold value of the nighttime light data. For example, a threshold based on DMSP-OLS data has been adopted by studies to qualitatively or quantitatively partition urban areas [51,52,53,54]. Based on this, taking reference from the studies of He et al. [55], Xu et al. [56], and Yang et al. [57], the DMSP-OLS data, NPP-VIIRS data, land surface temperature data, and normalized difference vegetation index (NDVI) data were used to efficiently extract urban areas in China at a 1-km spatial resolution from 2000 to 2015 using the stratified support vector machine method (Figure 2). Subsequently, Landsat Thematic Mapper (TM)/Enhanced Thematic Mapper Plus (ETM+) images were used to examine their spatial accuracy. The accuracy verification results show an average Kappa value of 0.66 and an overall accuracy of 95.20% [55]. Therefore, these datasets could be used to accurately represent urban expansion in China.

The CO_2_ emissions data were retrieved from the Open-Data Inventory for Anthropogenic Carbon dioxide (ODIAC) fossil fuel emission dataset from the Center for Global Environmental Research (http://db.cger.nies.go.jp/dataset/ODIAC/), National Institute for Environment Studies, which is committed to supporting global environmental research by monitoring the global environment, developing databases, operating supercomputers, and providing facilities for data analysis. The ODIAC first introduced the combination of nighttime light data and the emission/location profile of a single power plant to estimate the spatial range of CO_2_ emissions from fossil fuels with a spatial resolution of 1-km and a unit of t/km^2^. Currently, ODIAC includes several versions, such as ODIAC2013a, ODIAC2015a, ODIAC2016, ODIAC2017, and ODIAC2018. In this study, we used the ODIAC2016 data product, which was generated by combining multisource nighttime light data, the global point source database, and ship/aircraft fleet orbits [58] (Figure S1). The verification results clearly show that the ODIAC2016 data can effectively match the CO_2_ emissions at the global, regional, and city scales [58]. Therefore, the data product meets the requirements of large-scale and long time series [59]. Ultimately, we extracted urban CO_2_ emissions in China from 2000 to 2015 based on these datasets.

### 2.3. Potential Driving Forces

**Figure 3:**
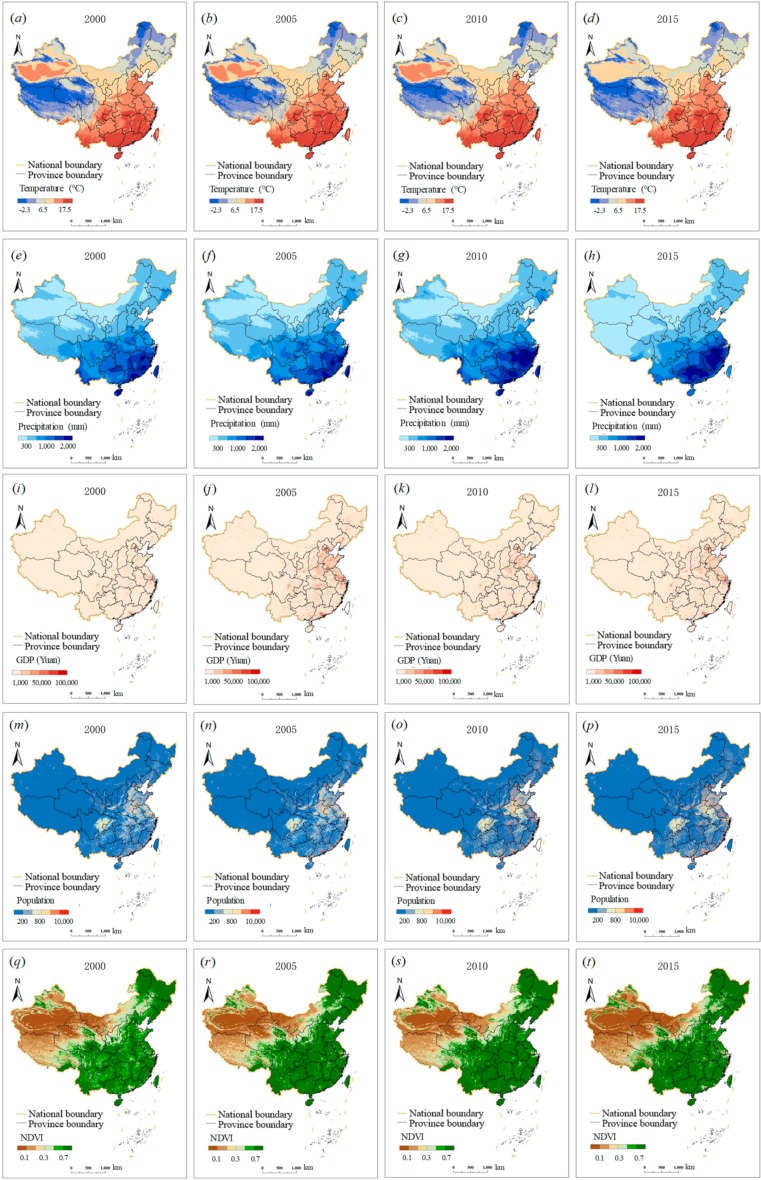
Spatial distribution of the potential driving factors in China from 2000 to 2015. Note: (**a**–**d**) temperature, (**e**–**h**) precipitation, (**i**–**l**) GDP, (**m**–**p**) population, and (**q**–**t**) NDVI.

In this study, we divided the potential driving factors into two categories: Socioeconomic factors and natural factors. Based on a literature review [17,60], six factors, namely, precipitation, slope, temperature, and NDVI, as natural factors, and population density and GDP, as socioeconomic factors, were selected to investigate the driving forces of urban CO_2_ emissions in China (Figure 3, Figure S2). All the data have passed collinearity tests, so each factor influences a different aspect of urban CO_2_ emissions [61].

Population density has been proven to be a significant factor driving urban expansion and CO_2_ emissions [62]. The 2000–2015 population data were obtained from the Data Center for Resources and Environmental Sciences, Chinese Academy of Sciences (RESDC) (http://www.resdc.cn) (Figure 3m–p). With regard to the population, some studies have found that population density has a significant positive effect on CO_2_ emissions and a negative spatial spillover effect [63], suggesting that regions with high population densities, such as China’s urban agglomerations, contribute more to environmental pollution [64]. Therefore, the population density of the areas under study could significantly affect urban CO_2_ emissions.

GDP is also a factor affecting urban CO_2_ emissions, as China is implementing industrial adjustments and transformation from traditional industry towards high efficiency, low-energy consumption levels [63]. The data in our study were acquired from the RESDC (Figure 3i–l).

The combination of temperature and precipitation is often referred to as climate, which is closely related to human production and habitation activities. Climate influences agriculture, industry, and energy supply, and cannot be ignored. Previous studies have reported the share of meteorological factors (temperature and precipitation) in different industrial sectors: 14.38% in the mining industry, 4.71% in the construction industry, and 8.20% in the manufacturing industry [65]. Because of their effects on industrial activities that are closely related to urban CO_2_ emissions, temperature and precipitation are indirect influencing factors on urban CO_2_ emissions. These data were also collected from RESDC (Figure 3a–h).

It can easily be seen that regions with flat terrain usually have advanced economic development in China (Figure S2). The slope affects urban CO_2_ emissions indirectly by influencing urban expansion and the economic boom. In this study, the slope was calculated using digital elevation model data. The data derived from the Shuttle Radar Topography Mission were downloaded from the Consortium for Spatial Information (CGIAR-CSI) (http://srtm.csi.cgiar.org/), which offers a major advance in the accessibility of high-quality elevations with 250 m spatial resolution.

NDVI was also used as an influencing factor to further analyze urban CO_2_ emissions. Vegetation growth has a great impact on CO_2_ emission concentrations, which in turn, react to environmental CO_2_ pollution. Thus, vegetation is also closely related to urban CO_2_ emissions. In this study, the 2000–2015 monthly NDVI composites were obtained from the Geospatial Data Cloud (http://www.gscloud.cn/). These data have been processed by systematic correction and given in a 1-km spatial resolution. We ultimately generated annual NDVI composites for 2000–2015 based on the average fusion (Figure 3q–t).

Ultimately, all of the spatial data were projected into an Albers conic equal area projection and resampled to a spatial resolution of 1 km.

### 2.4. Probit Model

Many mathematical models have been used to evaluate the driving forces of urban CO_2_ emissions, mostly via regression model. Traditionally, ordinary least squares (OLS) regression has been widely used to validate the relationships between urban CO_2_ emissions and potential driving factors [66]. The OLS regression has noteworthy limitations as a result of the assumptions that the error term is continuous symmetric and that the independent variable is linear. In many practical problems, the corresponding variable is not continuous, so the discrete choice model has been introduced here accordingly [67]. A discrete choice model (e.g., probit model), most frequently a binary choice model [68,69], could quantify the relationships between urban CO_2_ emissions and potential driving factors in pixels by binary data. The advantage of the binary selection model is that the results can directly predict locations that are likely to be urbanized [60]. The value of the response variable in the probit model is 0 or 1. The probit model has been applied in many fields, such as medicine, biology, and econometrics [67]. Thus, to explore the impact of each driving factor on urban CO_2_ emissions, the probit model was adopted in this study. The model can be expressed as follows:

(1) $$P\;\left(Y\;=\;1|X\right)\;=\;\varphi (\alpha \;+\;X\prime \beta )$$

The model is a binary response, nonlinear function, and *φ*(x) obeys the standard normal distribution, where *α* and *β* are parameters; *Y* = 1 means that the independent variable influences the dependent variable, and *X* indicates that each dependent variable has the same dimension as *X*.

To more clearly express the influence of each factor affecting urban CO_2_ emissions, we have incorporated temperature, precipitation, NDVI, slope, population, and GDP into Equation (1); the model can then be expanded into the following equation:

(2) $$P\;\left(Y\;=\;1|X\right)\;=\;\varphi (\overset{n}{\underset{i\;=\;0}{\sum }}\beta_{i}X\;+\;u)$$

where *βi* is the coefficient of the driving factor, n is the number of variables, and u is the interference residual value. The larger the absolute value of the coefficient, the more urban CO_2_ emissions will be affected by this factor; conversely, a smaller absolute value leads to a smaller effect. A positive value indicates a promoting effect on urban CO_2_ emissions, and a negative value implies a negative effect on urban CO_2_ emissions.

## 3. Results

### 3.1. Spatiotemporal Variations of Urban CO_2_ Emissions

**Figure 4:**
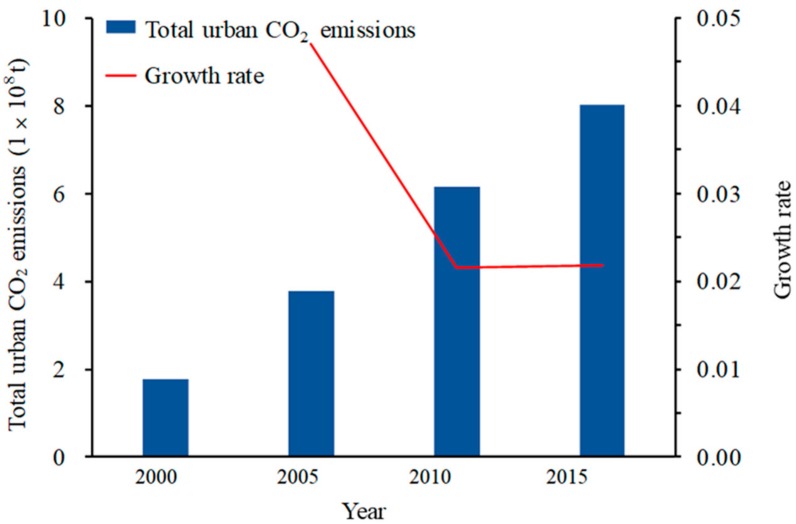
The total urban CO_2_ emissions and corresponding growth rate in China from 2000 to 2015.

As shown in Figure 4, total urban CO_2_ emissions showed a trend of increasing year by year, from less than 2 × 10^8^ t in 2000 to nearly 8 × 10^8^ t in 2015, which is four times the amount in 2000. From 2000 to 2005, total urban CO_2_ emissions doubled, reaching 4.5%. From 2006 to 2010, the growth rate was significantly lower than that of the previous stage, decreasing by 2.1%. From 2011 to 2015, the growth rate slightly increased and remained above 2.0%. One interesting phenomenon identified was that these three stages were basically consistent with the three stages of China’s energy strategy plan, e.g., the “Development Plan of New and Renewable Energy Industry in China for 2000–2015”. First, the plan established an economic incentive policy system and an industry management system from 2000–2005. The total annual exploited and utilized amounts of new and renewable energy only account for 0.70% of total commercial energy consumption. Hence, urban CO_2_ emissions always have a high rate. Second, the plan aimed to further improve the economic incentive policy system and the technological monitoring and servicing system for new and renewable energy from 2006-2010. The new and renewable energy percentage reached 1.25%, and correspondingly, the urban CO_2_ emissions rate exhibited a massive downturn, dropping to half of the original rate. Third, the plan called for new and renewable energy to become one of the new, important, emerging trades in China from 2011–2015. The total urban CO_2_ emissions rate remained at approximately 2.0%.

**Figure 5:**
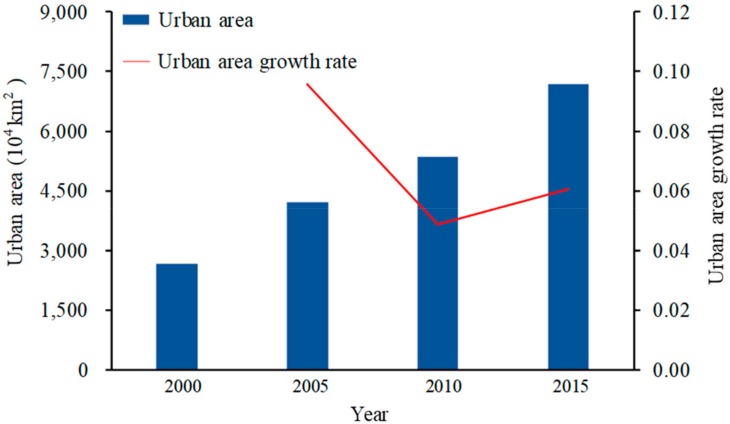
Urban area and corresponding growth rate in China from 2000 to 2015.

Through a comparison of Figure 4 and Figure 5, we found that from 2000 to 2015, with the expansion of urban area, the increase in urban CO_2_ emissions presented the same trend. Upon closer inspection, the urban area growth rate exceeded the urban CO_2_ emission growth rate; however, from 2005 to 2010, both rates declined greatly, although the urban area growth rate decreased more notably. After 2010, both rates were steady and again increased slightly. From the above findings, we could easily determine that urban CO_2_ emissions are accompanied by urban expansion, and the growth trends of the two variables are similar.

**Figure 6:**
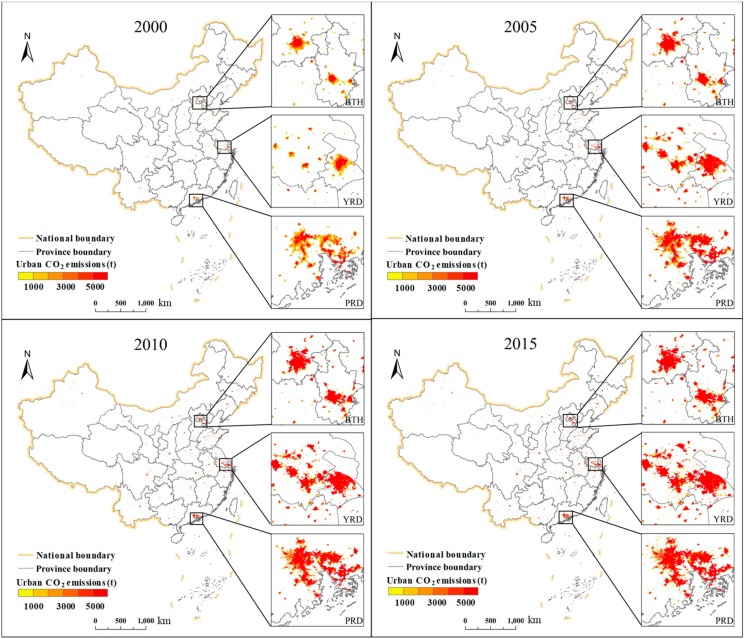
Urban CO_2_ emissions in China from 2000 to 2015. Note: The three magnified urban agglomeration areas are BTH, YRD and PRD. BTH represents the Beijing-Tianjin-Hebei urban agglomeration; YRD represents the Yangtze River Delta urban agglomeration; and PRD represents the Pearl River Delta urban agglomeration.

Spatiotemporal variations in urban CO_2_ emissions in China from 2000 to 2015 are shown in Figure 6. We found that urban CO_2_ emissions were concentrated in the six urban agglomerations. In 2000, for the BTH, urban CO_2_ emissions were mainly concentrated in the southeast region of Beijing and slightly to the south of the central region of Tianjin. Urban CO_2_ emissions in the YRD were mainly concentrated to the north of Shanghai and scattered within southern Jiangsu province and northeastern Zhejiang province. Urban CO_2_ emissions were mainly concentrated in the northeast and northwest of the PRD and far from the coastline. By 2005, in the YRD, Shanghai’s urban CO_2_ emissions extended from the north to the surrounding areas. In the MSL, urban CO_2_ emissions were concentrated in a continuous irregular area near Shenyang. The urban CO_2_ emissions of SP were mainly concentrated in the central region and had a sporadic distribution. In the CY, small patches of urban CO_2_ emissions were located in Chengdu and Chongqing. In 2010, urban CO_2_ emission concentration areas in the BTH, YRD, PRD, and MSL further expanded, the sporadic areas gradually became patches, and the patches gradually expanded. By 2015, the BTH, YRD, PRD, and MSL had steadily expanded, and the SP and CY had developed sporadically. Initially, the main areas generating urban CO_2_ emissions, in general, were a few concentrated regions, such as Beijing, Tianjin, Shanghai, Foshan, and Guangzhou, but urban CO_2_ emissions continuously expanded to the surrounding areas. Small areas expanded into larger areas and sporadic areas, and more small areas formed.

### 3.2. Results of the Driving Forces of Urban CO_2_ Emissions at the National Level

Table 1 shows the regression coefficients of driving forces that affect urban CO_2_ emissions using the probit model from 2000 to 2015. The effect of each factor on urban CO_2_ emissions is highly significant. Among these factors, NDVI (1.77), population density (0.14), GDP (0.12), and slope (0.02) were significantly positively correlated with urban CO_2_ emissions. In contrast, temperature (−0.01) and precipitation (−0.11) were significantly negatively correlated with urban CO_2_ emissions, respectively.

### 3.3. Results of the Driving Forces of Urban CO_2_ Emissions at the Urban Agglomeration Level

The driving forces of urban CO_2_ emissions at the urban agglomeration level are shown in Table 2. Urban CO_2_ emissions in the CY were notably influenced by every factor. Among the factors, temperature (−0.64), precipitation (−0.78), and slope (−0.04) had negative impacts, while population density (0.10), GDP (0.09), and NDVI (1.70) had positive impacts. In the BTH, slope, precipitation, temperature, population density, and NDVI had a remarkable influence, among which slope, population density, and NDVI had positive impacts on urban CO_2_ emissions with coefficients of 0.11, 2.37, and 2.19, respectively, while temperature (−0.10) and precipitation (−0.92) had negative impacts. In the MSL, slope, population density, NDVI, and GDP were significant influencing factors. Slope, population density, and NDVI had positive impacts, with coefficients of 0.11, 0.38, and 1.71, respectively, while GDP (−0.03) was a negative factor. In the SP, slope (0.29), population density (0.47), and NDVI (4.38) positively affected urban CO_2_ emissions, while GDP (−0.11) was a negative factor. In the YRD, six factors had significant impacts on urban CO_2_ emissions. Slope had a negative influence, and the other factors had positive influences. The coefficients were 0.42 (precipitation), −0.15 (slope), 0.04 (temperature), 0.51 (population density), 3.08 (NDVI), and 0.20 (GDP), respectively. In the PRD, all the factors (except for temperature) had positive impacts on urban CO_2_ emissions. The coefficients were 0.04 (precipitation), 0.32 (slope), 0.32 (population density), 0.99 (NDVI), and 0.07 (GDP), respectively.

## 4. Discussion

### 4.1. Driving Forces of Urban CO_2_ Emissions at the National Level

From Table 1, at the national level, we found that each factor had a significant impact on urban CO_2_ emissions. It should be noted that some driving factors had negative effects on urban CO_2_ emissions, such as temperature and precipitation, while others had positive effects, such as slope, population, NDVI, and GDP. In terms of temperature, China has a cold living environment in winter; thus, coal has become the main source for heating, especially in northern China, resulting in high CO_2_ emissions [70]. Precipitation, accompanied by winds, could relieve certain CO_2_ concentrations in the air, so more rain means less urban CO_2_ emissions [71]. Slope, population, GDP, and NDVI are all positive driving forces. An increase in population leads to an increase in man-made CO_2_ emissions; for example, the increase in population means more families and more private cars, which leads to an increase in urban traffic CO_2_ emissions [72]. The growth of GDP is also inseparable from the development of industry. Many factories emitted a large amount of CO_2_ [64]. In addition, the greater the degree of the slope, the less likely it is that CO_2_ produced in the region will spread, which might lead to an increase. However, the results of the NDVI factor did not seem to fit our expectations. We believe that vegetation could absorb CO_2_, but in this result, the more vegetation there was, the more urban CO_2_ emissions were observed. Because built-up areas were applied to extract the urban CO_2_ emissions and various potential factors, the vegetation captured here is urban vegetation. While vegetation has an absorption effect on CO_2_, relative to the large amounts of urban CO_2_ emissions, the vegetation’s absorption effect is not substantial. Therefore, NDVI shows a positive impact on urban CO_2_ emissions [73]. Moreover, NDVI is generally located near residential or industrial areas, meaning that it is often accompanied by residential or industrial CO_2_ emissions, perhaps forming the false impression that urban CO_2_ emissions have a positive correlation with the growth of NDVI [66].

### 4.2. Difference in the Driving Forces of Urban CO_2_ Emissions in the Six Urban Agglomerations

The study of various driving factors at the national level has neglected regional characteristics. Thus, the various driving factors on urban CO_2_ emissions were evaluated and compared at the urban agglomeration level. Table 2 shows each factor that affected urban CO_2_ emissions at the urban agglomeration level.

First, considering GDP, the highest degree of influence is seen in the YRD, with an influence coefficient of 0.20, while the lowest degree of influence is in the PRD, with a coefficient of 0.07. As is well known, the YRD and the PRD region are economically-developed areas of China, but from the results, we found a large difference in the degree of influence of GDP in the two urban agglomerations on CO_2_ emissions. In 2000–2015, the YRD was ranked at the top in China for secondary industry, which makes the greatest contribution to GDP accounts; however, a large number of factories were constructed in the development of secondary industry, causing high CO_2_ emissions. Therefore, GDP has a high influence in the YRD. By contrast, the PRD also has a high-level economy, but the degree of influence of the GDP is relatively small. The reason may be that the PRD’s tertiary industry is more advanced; thus, tertiary industry makes the greatest contribution to GDP. Tertiary industry represents the service industry, which emits less CO_2_ than secondary industry. Therefore, it is clearly indicated that the industry structure remains to be further improved, enhanced, and upgraded in the YRD.

Second, for the NDVI, we found that the NDVI has a far higher influence in SP than in the other urban agglomerations, with a coefficient of 4.40. This phenomenon might be explained by the fact that, as shown in Figure 4, the distribution of vegetation in SP has an aggregation effect and is evenly distributed throughout, unlike the other urban agglomerations. Therefore, in the local industrial production area, the influence degree of the NDVI around factories would be relatively higher. In addition, based on the current situation wherein the built-up area in SP is dispersed, cities in SP are not very close to each other, and the central cities do not play a strong leading role.

Third, with regard to the driving factor of population density, we found that the degree of influence in BTH and SP is high, with coefficients of 2.37 and 2.47, respectively. Further analysis revealed that although population density has a high degree of influence in the two urban agglomerations, the reasons are different. The BTH has a large number of national high-tech industries and attracts many highly-competent people, forming a very large rainbow absorption effect that attracts many ordinary people from across China. The majority of these people are rural workers, and most of them provide supporting services [74]. Therefore, a large proportion of the migrant population is engaged in low-level services. The result of this influx is a large population, leading urban CO_2_ emissions to sharply increase in areas due to transportation and growth in the residential sector. However, for the SP, the effect of population density may be due to its large population base and heavy industry. Thus, the impact of the population factor on urban CO_2_ emissions is very notable.

Fourth, the slope is a negative driving factor in the CY and the YRD. The CY is located in a mountainous area. Compared to flat areas, high-altitude and steep areas are less likely to be developed because it costs more to construct built-up areas [75]. At the same time, ecological protection policies are adopted in areas with high gradients, and most have protected soil and water [66]. Thus, tourism is the prioritized development industry. Therefore, for the CY, the higher the slope, the less construction there is, and the less urban CO_2_ emissions; hence, the slope is a negative factor. For the YRD, protection and development policies are also implemented for areas with higher slopes [76]. Tourism and characteristic agriculture are also carried out in the region to increase local revenue. This situation provides a development concept for mountainous and hilly areas to make full use of the regional advantages and develop characteristic industries.

### 4.3. Difference in the Driving Forces of Urban CO_2_ Emissions at the Two Levels

Hierarchy and scale effects existed widely in all fields of socioeconomic development [17]. Due to China’s hierarchical administrative levels, a higher administrative level (e.g., China or urban agglomerations) generally has stronger administrative powers [44,77], consequently resulting in different spatiotemporal patterns and driving forces for urban CO_2_ emissions across different administrative levels. In this study, the model results suggest that regardless of which level was adopted, the impacts of the driving factors on urban CO_2_ emissions were quite different at the national and urban agglomeration levels.

Temperature and precipitation showed negative impacts at the national level, with similar impacts in the CY and the BTH. However, temperature and precipitation positively affected urban CO_2_ emissions in the YRD and the PRD. These two urban agglomerations are located in regions where the temperature and precipitation are relatively constant throughout the year; for example, residents in the YRD and PRD regions do not need to burn coal for heat in winter. Such climatic conditions are more conducive to productive activities. Therefore, temperature and precipitation are positive factors. Slope has a positive impact on urban CO_2_ emissions. Because built-up areas are almost always distributed in flat terrain at the national level, a low slope is beneficial for socioeconomic development. However, for CY and the YRD, the built-up areas are very hilly, which might negatively influence the two urban agglomerations. In addition, it is clear that the population density and GDP both have a positive impact on urban CO_2_ emissions at the national level.

We also found that all driving factors were significant at the national level. However, for urban agglomerations, there were multiple conditions that amplify the effects. Although the conditions are complex, they always have intrinsic causes. For example, with regard to temperature and precipitation, the PRD has a subtropical-tropical humid monsoon climate that remains stable and results in high temperatures and high rainfall year round [78], so that the climate has only a slight effect. For the BTH, GDP is not a significant factor; this region’s industry structure is multifaceted and multilayered [74], and the finance sector and high-tech industries account for the largest proportion of the GDP. Therefore, although GDP is high, it has a small impact on CO_2_ emissions.

The comparison of the national and urban agglomeration levels shows that the degree of influence at the national level is usually lower than that at the urban agglomeration level. On the one hand, for the various regions, the six factors (temperature, precipitation, GDP, slope, NDVI, and population density) have different dimension effects. However, the influence degree of each factor at the national level depends on the influence degree of that factor at the regional level. Therefore, the effect of each factor at the national scale may not be as great as that at the level of a small region of urban agglomeration [61,79]. Although the degree of influence of the factors on urban CO_2_ emissions was usually lower at the level of a single urban agglomeration, there are exceptions, because each urban agglomeration has different conditions than the national region.

### 4.4. Limitations and Future Directions

There are several limitations that are worth mentioning. As a complex environmental problem, many other factors may affect urban CO_2_ emissions, and many aspects should be further studied, such as agricultural and construction factors [80]. The selected factors are mostly natural potential forces, but socioeconomic factors also play a significant role, such as transportation, distance from a body of water, built-up area, and residential emissions. These factors should be incorporated into the study. In addition, with the development of urban agglomerations, the regions are changing, and the number of urban agglomerations will continue to rise; therefore, we should update the urban area data over time by refining the spatial resolution from 1000-m to 500-m, even at the larger scale. The images captured by sensors on high-resolution satellites, such as Landsat series images, can be used to more accurately interpret urban areas. The model in this study is an economic one that did not consider spatial locations, and therefore, the impact of the spatial location was not examined; hence, the applicability of the geographic data needs to be improved. Other appropriate models could also be used, such as the panel model, which has been improved with regards to capturing undesirable environmental outputs; in addition, panel data models [81], static and dynamic panel models [82], panel cointegration models [83], and modified input-output models could be used [84].

## 5. Conclusions and Policy Implications

This study explored the driving forces of urban CO_2_ emissions in China with a comparative analysis between national and urban agglomeration levels. We selected four years—2000, 2005, 2010, and 2015—to clearly determine the total amount and rate of urban CO_2_ emissions growth by analyzing the spatial and temporal changes in urban CO_2_ emissions from 2000–2015. Temporally, it was observed that the total urban CO_2_ emissions have consistently increased from 2 × 10^8^ t to 8 × 10^8^ t, but the rate of increase drastically declined after 2005 and then stabilized, which corresponds with the implementation of the policies outlined in the “Development Plan of New and Renewable Energy Industry in China for 2000–2015”. A probit model was used to quantify the effects of six factors (population density, GDP, slope, temperature, precipitation, and NDVI) on urban CO_2_ emissions. At the national level, a cold living environment in China in winter might play an important role in promoting coal burning and heating, and thus, the temperature has a negative impact on urban CO_2_ emissions. The NDVI is an interesting positive factor and is an indicator of the degree of urban CO_2_ emissions. Vegetation in cities is limited, and although a large proportion of urban CO_2_ emissions could be absorbed, vegetation only slightly affects the amount of urban CO_2_ emissions. At the urban agglomeration level, we observed certain phenomena and examined the results in detail. We found that even though the YRD and PRD are both economically-developed regions with GDPs among the highest in China, the influence of GDP on urban CO_2_ emissions in the YRD and PRD are vastly different. GDP has the highest degree of influence in the YRD and the lowest degree of influence in the PRD. Upon closer examination, the YRD and the PRD have different industry structures, with the YRD having many secondary industry enterprises that are mainly engaged in manufacturing, resulting in the area being at the top in China. However, the GDP percentage of tertiary industry in the PRD is higher than that in the YRD. The comparison of the two urban agglomerations reveals that the YRD should readjust and upgrade its economic structure. In SP, the data show that vegetation has a cumulative effect that is evenly distributed throughout SP; the vegetation in built-up areas does not absorb a large proportion of the urban CO_2_ emissions. The high coefficient indicates that SP still has many CO_2_-intensive industries. Given the scattered built-up areas, it is not difficult to observe that the cities in SP are not close to each other, which further explains that the central city does not play a strong driving role. Moreover, the population density factor has a high degree of influence on the urban CO_2_ emissions in BTH and SP, but there are again different reasons. In BTH, a large number of people engage in low-level service employment, and in terms of transportation, a high number of residents will cause high CO_2_ emissions. At the same time, in BTH, the population is mainly concentrated in Beijing and Tianjin. For mountainous areas, the slope factor negatively affects the urban CO_2_ emissions in CY and the YRD; the higher the slope, the lower the CO_2_ emissions.

We also compared the results at the national and urban agglomeration levels. In contrast to the national level, the climate is a positive factor in the YRD and the PRD. At the national level, each factor is significant, but for a single urban agglomeration, because of the characteristics of the region itself, the situation is more complex. The degrees of influence of most factors at the national level are lower than at the urban agglomeration level.

According to the results, several policy proposals are presented. First, the government policy-making department should introduce and support the development of new and renewable energy industries to effectively decrease urban CO_2_ emissions while contributing to the national economy. Spatially, we found that urban CO_2_ emissions are concentrated in the central cities of the urban agglomerations; thus, for the central cities, relevant governmental departments should actively adjust and upgrade the industry structures from traditional industries, such as low-level service industry, heavy industry, and manufacturing and handicraft industries, to intensively develop high-level service industries, high and new technology industries, and the finance sector. Moreover, we should learn from the development of central cities, and the central cities should guide the new and rapidly-developing cities.

Second, the YRD should readjust and upgrade its economic structure to increase its growth. In the SP, the government should intensively develop new, high-technology industries. In addition, because the SP has a long coastline and good harbors, the marine industry can be further developed. We believed that the SP should develop multiple industries based on its advantages. In the future, the Shandong government should foster the exchange of ideas and cooperation among cities and expand the leading role of the central cities. In the BTH, to mitigate the population pressure of the two largest cities, the government should intensively develop the surrounding areas, such as the cities in Hebei in the BTH. In the CY, the government should make full use of its regional advantages to develop characteristic industries.

Third, China’s government should establish a better regulatory system to coordinate urban CO_2_ emissions across the various regions, determine the problems that most affect the regions based on the available data, and develop national urban CO_2_ emission reduction guidelines to guide local governments to formulate specific policies according to the actual context.

## Figures and Tables

**Table 1:** Results of the probit model at the national level from 2000–2015.

**Coefficient**	**Precipitation**	**Slope**	**Temperature**	**Population Density**	**NDVI**	**GDP**
	−0.110 ***	0.023 ***	−0.013 ***	0.138 ***	1.765 ***	0.116 ***
Number	778,332	Log-likelihood	−102,850	Pseudo-R^2^	0.046	-

Note: Significant at the *** 1% level.

**Table 2:** Results of the probit model at the urban agglomeration level from 2000–2015.

Variable	CY	BTH	MSL	SP	YRD	PRD
Precipitation	−0.780 ***	−0.916 ***	0.166	0.000	0.415 ***	0.042 *
Slope	−0.036 ***	0.114 **	0.113 ***	0.288 ***	−0.147 ***	0.320 ***
Temperature	−0.637 ***	−0.096 ***	−0.012	0.000	0.037 ***	0.005
Population density	0.101 ***	2.365 ***	0.376 ***	2.469 ***	0.506 ***	0.316 ***
NDVI	1.698 ***	2.188 ***	1.713 ***	4.375 ***	3.079 ***	0.987 ***
GDP	0.085 ***	0.011	−0.032 *	−0.114 ***	0.196 ***	0.072 ***
Number	33,114	78,092	23,385	63,111	157,700	101,720
Log-likelihood	−2747.630	−2594.031	−1802.104	−1,735.822	−26,426.693	−15,238.895
Pseudo-R^2^	0.188	0.238	0.050	0.282	0.125	0.027

Note: Significant at the * 10% level, ** 5% level, and *** 1% level. BTH represents the Beijing-Tianjin-Hebei urban agglomeration; MSL represents the middle and south Liaoning urban agglomeration; SP represents the Shandong Peninsula urban agglomeration; CY represents the Chengdu-Chongqing urban agglomeration; YRD represents the Yangtze River Delta urban agglomeration; and PRD represents the Pearl River Delta urban agglomeration.

## Supplementary Materials

The following are available online at https://www.mdpi.com/1660-4601/16/19/3692/s1. Figure S1: CO_2_ emissions in China from 2000 to 2015, Figure S2: The slope of China.
