# Supplementary material for: What Are the Driving Forces of Urban CO2 Emissions in China? A Refined Scale Analysis between National and Urban Agglomeration Levels
Source: Int J Environ Res Public Health. 2019 Sep 30;16(19):3692. doi: 10.3390/ijerph16193692 (PMC6801949; doi:10.3390/ijerph16193692)
Supplement: Supplementary file 1 [file ijerph-16-03692-s001.pdf]

Support Information for

What Are the Driving Forces of Urban CO_2 Emissions in China? A Refined Scale Analysis between National and Urban Agglomeration Levels

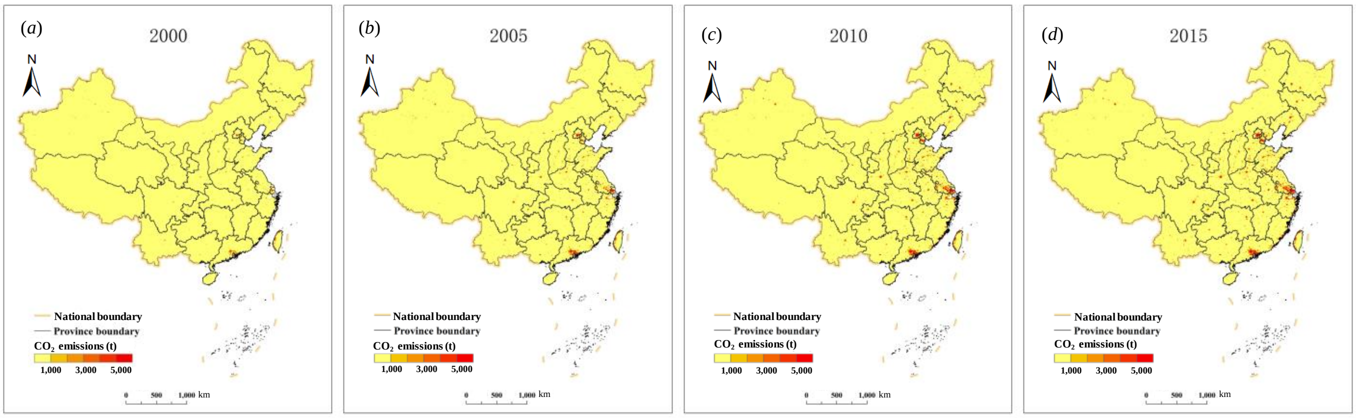

Fig. S1. CO_2 emissions in China from 2000 to 2015.

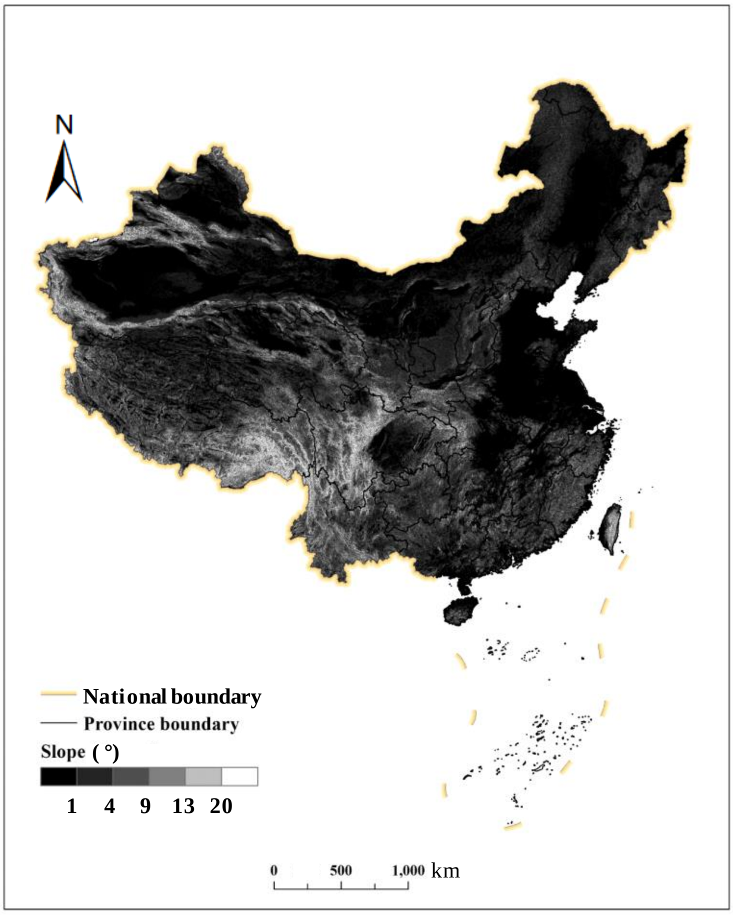

Fig. S2. The slope of China.
